# Advanced Applications of Silk-Based Hydrogels for Tissue Engineering: A Short Review

**DOI:** 10.3390/biomimetics8080612

**Published:** 2023-12-15

**Authors:** Zekiye Akdag, Songul Ulag, Deepak M. Kalaskar, Liviu Duta, Oguzhan Gunduz

**Affiliations:** 1 Center for Nanotechnology Biomaterials Application and Research (NBUAM), Marmara University, Istanbul 34890, Turkey; zekiyeekdg@gmail.com; 2 Division of Surgery Interventional Science, University College London, Royal National Orthopaedic Hospital, UCL Institute of Orthopaedic Musculoskeletal Science, Stanmore, London HA7 4LP, UK; songul.ulag@marmara.edu.tr (S.U.); d.kalaskar@ucl.ac.uk (D.M.K.); 3 Spinal Surgery Unit, Royal National Orthopaedic Hospital NHS Trust, Stanmore, London HA7 4LP, UK; 4 Lasers Department, National Institute for Laser, Plasma and Radiation Physics, 409 Atomistilor Street, 077125 Magurele, Romania

**Keywords:** silk-based hydrogels, tissue engineering, wound repair, drug delivery, biomimetics

## Abstract

Silk has been consistently popular throughout human history due to its enigmatic properties. Today, it continues to be widely utilized as a polymer, having first been introduced to the textile industry. Furthermore, the health sector has also integrated silk. The *Bombyx mori* silk fibroin (SF) holds the record for being the most sustainable, functional, biocompatible, and easily produced type among all available SF sources. SF is a biopolymer approved by the FDA due to its high biocompatibility. It is versatile and can be used in various fields, as it is non-toxic and has no allergenic effects. Additionally, it enhances cell adhesion, adaptation, and proliferation. The use of SF has increased due to the rapid advancement in tissue engineering. This review comprises an introduction to SF and an assessment of the relevant literature using various methods and techniques to enhance the tissue engineering of SF-based hydrogels. Consequently, the function of SF in skin tissue engineering, wound repair, bone tissue engineering, cartilage tissue engineering, and drug delivery systems is therefore analysed. The potential future applications of this functional biopolymer for biomedical engineering are also explored.

## 1. Introduction

Scientists have not determined a specific date for the first discovery of silk, but it is confirmed by many sources that it was first used in China. The value of silk, initially utilized solely for textiles [1], continues to hold significance in today’s textile industry [2,3]. Beyond its widespread use worldwide, silk has undergone further modifications in the field of medicine [4] and has been employed as sutures [5]. As the research on silk has increased lately, its application areas have also expanded towards other important fields such as filtering systems [6], electronics [7], photonic applications [8], drug delivery systems [9], nanotechnology [10], and tissue engineering [11].

One should emphasize that hydrogels exhibit a structural resemblance to the natural extracellular matrix (ECM), which distinguishes them from other materials. This similarity allows hydrogels to emulate the properties of natural tissues, rendering them highly appealing for applications in regenerative medicine, particularly in the context of soft tissues [12,13]. Over the past decade, in the field of biopolymers used for the fabrication of hydrogels, silk fibroin (SF), a natural polymer approved by the Food and Drug Administration, has gained increased attention. Thus, SF derived from the *Bombyx mori* (*B. mori*) silkworm cocoon represents a commonly utilized biomaterial in the fields of tissue engineering and regenerative medicine (i.e., corneal and nerve regeneration [13,14,15], or as a wearable sensor [16]. This is mainly due to its demonstrated biocompatibility, biodegradability, versatility of structural adjustment, and the possibility to tailor the mechanical characteristics. In addition, considerably abundant SF sources with low production costs are available [16]. Unfortunately, SF brittles easily because of its relatively simplistic structure, which drastically limits its potential usability. To overcome this drawback, one emerging strategy was to blend SF with various natural (i.e., gelatine, collagen, alginate, and hyaluronic acid) or synthetic (i.e., polyvinyl alcohol, polyethylene, and polyurethane) biopolymers [17,18].

SF is generally dissolved in water-based solvents, enabling its facile reconstruction into diverse material formats such as sponges, hydrogels, mats, and/or films. This versatility is easily achieved through the application of different fabrication methods, including but not limited to physical-chemical crosslinking [19], freeze-drying [20], electrospinning [21], 3D bioprinting [22], and spin coating [23]. In contrast to chemical and enzymatic crosslinking methods, photo-crosslinking offers several advantages, including the generation of fewer by-products, excellent biocompatibility, high efficiency, and precise controllability [24]. A quick and effective method used to produce evenly continuous fibers with nano- to micro-sizes is electrospinning [25]. The morphology of electro-spun fibers, with micro- and nano-dimensions, can enable exudate absorption and regulate the adaptation and proliferation of dermal cells. Additionally, it facilitates efficient gas exchange in the wound bed [26]. These micro/nanofibers, which come in the shape of nonwoven fibers, are extensively applied in the fields of biomaterials, tissue engineering, and drug delivery [27]. Specifically, optimal electrospinning processes can further produce nanostructured fibers with remarkable flexibility [28]. Freeze-drying, also known as lyophilization, is a versatile technique that facilitates both the shaping of natural materials into specific geometries with a porous microarchitecture and the customization of their structural and biological characteristics [29]. A bottom-up additive fabrication process called 3D printing creates structures layer by layer, covering multiple length ranges with excellent precision. This method facilitates the generation of intricate geometries that are hard to manufacture or impossible to produce by traditional methods [30]. Direct printing of human organs is made more feasible by bio-inks, which were demonstrated to enclose and protect stem cells during the 3D printing process [31].

This work includes a brief introduction to SF and a literature overview utilizing a variety of methods to enhance the tissue engineering of hydrogels based on SF. A particular emphasis is given to the functional role of SF in skin tissue engineering, wound repair, bone tissue engineering, cartilage tissue engineering, and drug delivery systems. Potential future applications of this polymer in biomedical engineering are also discussed.

## 2. Silk Fibroin Use for Various Biomedical Applications

### 2.1. Silk Fibroin

**Figure 1:**
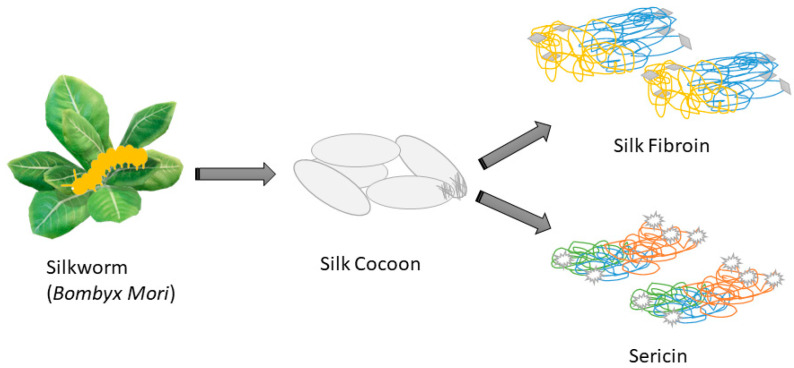
Proteins inside silk cocoons are produced by *Bombyx mori*.

Silk is obtained from both spider webs and silkworms. The acquisition of silk from spiders poses more difficulties as spiders are wild animals, and the sustainability of web formation is more restricted. Most of the SF reported in the literature has been derived from a species of silkworm cocoon known as *Bombyx mori*. SF comprises three primary proteins, namely one P25, one light chain, and one heavy chain [8], present in a ratio of 1:6:6, respectively [32]. The hydrophobic heavy chains exhibit a molecular weight of 391.367 kDa, 5263 amino acid residue lengths, and an isoelectric point of 4.22. These chains predominantly consist of 45.9% glycine, 30.3% alanine, 12.1% serine, 5.3% tyrosine, 1.8% valine, and only 4.7% of the other 15 amino acid species [8] (Figure 1). The primary structure of the hydrophobic heavy chain contains a low-complexity region that is highly repetitive and flanked by N- and C-termini. This region of the chain contains a repetitive sequence dominated by GAGAGS (432 copies) and GAGAGY (120 copies), which account for 72% of the low-complexity region [22]. The light chain is hydrophilic [8], 244 amino acids long, and has a molecular weight of 25.8 kDa [33]. In comparison to the heavy chain, the light shackle has a separated amino acid combination and fewer non-repeating sequences. Hydrophilic light chains consist of 14% alanine, 9% glycine, 10% serine, and serine-acetylated N-terminal residues [8]. Despite being lightweight, its tensile strength is significantly superior to that of other biopolymers and comparable to that of nylon and mild steel [34,35]. The heavy and light chains form a complex through a disulfide bond [36] between Cys-20 of the heavy chain and Cys-172 of the light chain, creating a heavily-light chain structure [33]. Another major protein structure, P25, is a glycoprotein (MW: 30 kDa). The function of P25 is to establish the stability of the heavy-light chain coordination compound through hydrophobic interactions with the heavy chain [8].

The composition of SF primarily consists of 72–81 wt.% fibroin, 19–28 wt.% sericin, 0.8–1 wt.% oil/wax, and 1–1.4 wt.% dye. Fibroin, the constituent with the highest concentration, provides mechanical strength, whereas sericin possesses an adhesive structure, assisting in holding the overall structure together [37]. Despite their small diameter, silk fibers interlock strongly due to interaction with each other, resulting in a diameter increase of up to 20–200 nm [38]. Additionally, SF demonstrated exceptional thermal stability, maintaining its integrity up to approximately 200 °C. Beyond this temperature, the cleavage of side groups of amino acids and the dissociation of peptide bonds occur [39].

At the macroscopic level, the form of the *B. mori* silk fibers is composed of an inner fibroin layer and an outer sericin coating resembling a core shell. The fibroin core of *B. mori* silk comprises two brins, which exhibit a trilateral cross-section. The sericin layer has a thickness in the range of 0.5–2 microns. The size of the silk thread alters depending on the silkworm’s genus and species. *B. mori* silk fibers contain sericin, which has diameters ranging between 10 and 25 μm. At the microscopic scale, the arrangement of these fibers displays a hierarchical construction in which well-organized antiparallel β-sheet nanocrystals are dispersed within a semi-amorphous matrix of helices and β-turns, loosely aligned with the fiber axis. In the degummed fiber, the compactly H-binded β-sheet nanocrystals, which make up 50% to 60% of the volume percentage of fibroin, produce higher tensile strength and modulus [40]. Additionally, the restriction of β-sheet nanocrystals and other semi-amorphous arrangements at the nanoscale in silk fibers is crucial for achieving high mechanical strength. To bridge the space between various β-sheet nanocrystals, uncrystallized molecular chains adopting random coil, helical, or β-turn shapes must undergo substantial deformations [8].

Numerous studies in the dedicated literature have emphasized that silk possesses exceptional mechanical properties, including remarkable strength and durability, within the domain of materials science. Such properties undoubtedly offer unlimited opportunities to produce robust and durable biomaterials due to their functionalization and ease of processing. Collagen (COL) and polylactic acid (PLA), which have demonstrated toughness, offer superior stiffness properties compared to frequently used biomaterials, along with favorable polymeric degradability. Silk obtained from silkworms is a more durable material than synthetic polymers, including polyurethane and polystyrene. Additionally, silk is biodegradable, and its rate of degradation varies based on the processes it undergoes. The increase in β-sheet ratio in silk’s structure impacts the degradation rate in the opposite direction [8].

Silks are examples of highly effective biomaterial designs because they are tailored hierarchical nanostructures with remarkable mechanical qualities [41]. These biomaterial designs encompass primary coatings, state-of-the-art biosolids, and advanced vaccines for various biomedical applications. Enhancing these matrices by combining functional properties like conductivity or delivery of bioactive molecules or compounds (i.e., growth factors, drugs, antibiotics, genes, or cells) makes them intelligent matrices for skin tissue regeneration and repair [42]. Furthermore, the capacity of silk to absorb moisture is another noteworthy feature that proves beneficial in clinical applications, besides its antimicrobial and anti-irritation characteristics [42].

D’Antuono et al. highlighted the structural similarity between silk and the stratum corneum, which is the outermost layer of the skin. Since silk shares properties similar to those of human skin, such as functioning as a barrier, it can replicate several positive effects on the skin [41]. Therefore, silk holds the potential to be an efficient component in the creation of artificial leather.

Moreover, SF possesses properties, such as spinnability and cell compatibility, that have allowed it to be utilized as a bioink in 3D printing and spinning technologies [42,43]. In 3D applications involving extrusion, the interactions of hydrophobic structures in the protein sequence, induced by pressure, lead to the gradual transition of a liquid solution into a solid state. During spinning, both the rheological properties of the solution and the degradation of the SF structure occur [44]. SF is frequently combined with various compounds (Table 1), including gelatine [45], alginate [46], PCL [47], and PLA [48], to create bio-inks [49]. Physical, chemical, ionic, or photo-crosslinking mechanisms are typically used to stabilize printed scaffolds. Alternatively, stabilization may occur after printing by submerging the scaffold in a support bath [49,50].

Due to its bond structure and conformation, there has been an effort to convert SF into a gel form using a photo-crosslinking agent. The outcome of these experiments was the successful synthesis of methacrylate SF (Sil-MA), a novel product that exhibited superior mechanical strength and improved cell biocompatibility, proliferation, and differentiation in comparison to SF alone [51,52].

**Table 1:** Examples of some biomaterials used in the silk-fibroin combination.

SF-Based Hybrid Biopolymers	Concentration of Biomaterials	Production Method	Application Area	Cell Types	Results	Ref.
SF/Gelatin	5% *w*/*v* SF 5% Gelatin	Extrusion-based 3D bioprinting	-	hMSCs	Cell viability up to the 28th day after printing	[53]
SF/Gelatin	5% *w*/*v* SF 5% Gelatin	3D bioprinting	Bone	hMSCs	Osteogenic differentiation at gene and protein levels A novel in vitro model that replicates a dimensional microenvironment.	[54]
SF/Gelatin	5% *w*/*v* SF 5% Gelatin	3D bioprinting	Skin	Human primary adult dermal fibroblasts	Crossinkable in situ Printability as bio-ink	[55]
Sil-MA	30% *w*/*v* Sil-MA	Digital Light Processing (DLP)	Trachea	NIH/3T3 fibroblast	Excellent biocompatibility, swelling behavior, cell growth, Availability of multi-layer printing	[51]
Sil-MA	15% and 30% Sil-MA	4D bioprinting	Trachea	Chondrocytes and turbinate-derived mesenchymal stem cells	Reproducibility of heterogeneous tissues containing more than two components	[56]
SF/Alginate	5% *w*/*v* SF 5 wt.% Alginate	3D printing	Microchannel network	NIH/3T3 fibroblast	Good cytocompatibility	[57]
SF-Polypyrrole	20 wt.% SF 14 mM pyrrole	3D printing and electrospinning	Neural tissue	Schwann cells	Improvement in bioactivity and mechanical strength with SF	[58]
SF/COL	COL:SF 2:1	3D bioprinting	Repairing of injured spinal cord	Mesenchymal stem cells	Enhanced multi-step stiffness	[59]
Cellulose NPs-reinforced chitosan/SF	5% *w*/*v* chitosan 1% *w*/*w* SF 1% *w*/*w* cellulose NPs	3D printing	Bone	Raw 264.7 cells	Enhancing osteogenic efficiency	[60]
SF-bioactive glass	6% *w*/*v* SF 6% *w*/*v* Gelatin 0.1%, 1%, and 10 wt.% bioactive glass	3D printing	Bone	Human bone marrow stem cells	Excellent mechanical stability	[61]
SF/PCL	16% *w*/*v* SF	3D printing	Meniscus regeneration	Synovium-derived mesenchymal stem cells	One-step operation Cost effective	[62]
SF-Decellularized ECM	5% *w*/*v* SF 8 wt.% Gelatin 10% Decellularized liver ECM	3D printing	Liver regeneration	Huh7 cells	Good cell viability	[63]
Polyethylene glycol di-methacrylate (PEGDMA)/SF	10 wt.% PEGDMA 8% *w*/*v* SF Ratio = 1:1	DLP 3D printing	Articular cartilage	-	More and small porosity	[64]
SF/Gelatin	0.12, 0.16, 0.2 wt%	Diffusion-driven cross-linking	Peripheral nerve repair	Schwann (SC) and PC12 cells	Myelination of SC, neuronal differentiation of PC12 cells	[15]
SF/Gelatin/Chitosan	3.5% *w*/*v* SF 1:3 *v*/*v* Chitosan:gelatin	Freeze-drying	Tissue engineering	Human umbilical vein endothelial cells	Suitable pore size, pore interconnectivity, porosity, increased mechanical strength, and degradation rate	[18]

It is important to mention that SF is used in a diverse range of fields, such as skin injuries, wound healing, bone and cartilage injuries, and drug delivery systems. In the next sections, a selection of the results reported on these types of applications will be introduced and discussed.

### 2.2. Silk Fibroin in Skin and Wound Tissue Engineering

The skin is the largest organ in the human body (approx. 7%) [65], and it is responsible for immunological, protective, and sensory functions [66]. Due to its exposure to the external environment, it is one of the body parts that primarily undergoes changes in terms of tissue loss, regeneration, and differentiation. The skin is essential for connecting the external environment to the inner workings of the body [67]. One of the paramount approaches to wound repair is the successful mimicry of the ECM [68] and the facilitation of angiogenesis [69]. It is vital to treat skin injuries promptly and efficiently, with minimal harm and without the risk of complications [70]. The primary goal is to restore the wound tissue’s histology and physiology while achieving successful repair and treatment [71]. Various methods are being discovered for tissue engineering and regenerative medicine approaches.

There are extensive reports in the literature on the use of SF in skin tissue engineering and wound repair, with promising results [72]. Some of the reasons why SF is preferred for use in wound repair could be: (i) silk protein’s high moisture retention capacity [71], (ii) its ability to establish a good connection between the produced biomaterial and tissue cells [73], (iii) its lack of allergic effect [74], (iv) its neovascularization for rapid repair of damaged tissue [75], (v) its ability to increase re-epithelialization in tissues, (vi) its easy production and use [76], and finally (vii) its easy adaptability to different materials, additives, and polymers to produce hybrid composite structures [77].

Turkkan et al. developed 3D porous scaffolds for skin tissue engineering therapy using SF-functionalized citrus pectin (fPEC). The SF provided mechanical strength to the scaffolds, while the fPEC increased their structural stability and water affinity. After freeze-drying, the air plasma treatment removed the polymer skin layer from the scaffolds. After conducting the requisite analysis and characterization tests, it was determined that the porous nature of the SF/fPEC scaffolds proved to be suitable for fibroblast cell attachment, proliferation, and migration toward a 3D matrix, exhibiting promise as a material for skin substitutes in dermal regeneration [78].

Bhardwaj et al. fabricated a bioactive scaffold containing 2% keratin and 2% SF in its composition. The composite scaffold was determined to be suitable for dermal tissue reconstruction as it effectively supported the fundamental requirements for fibroblast growth, attachment, and proliferation. Moreover, it facilitated the deposition of intact ECM (specifically collagen type I), further indicating its potential for various in vitro skin tissue engineering applications [79].

Keirouz et al. employed nozzle-free electrospinning, a unique technique divergent from existing electrospinning methods. A distinguishing aspect of this technique is the absence of a needle; instead, it utilizes jet formation from multiple locations to produce fibers at a quicker rate. Using this method, the authors successfully fabricated SF-based composite fibers with customizable wettability at various concentrations. The fibers’ ability to create a wound dressing that aids the healing of skin tissue and the absorption of exudate was therefore highlighted [80].

Ju et al. studied the preparation of SF nanomatrices by electrospinning and evaluated their use as a wound dressing material in a rat burn model. After a 14-day period, the wounds treated with the SF nanomatrix displayed a morphology and histology akin to normal skin. Therefore, complete regeneration of the wound area and an absence of edema or granulation tissue formation were observed. In contrast, the group treated with medical gauze (control) exhibited persistent infiltration of neutrophils and lymphocytes. By day 28, the wound areas treated with the SF nanomatrix had significantly reduced to 4% of their original size, while the control group still maintained a wound size of 18%. Notably, collagen deposition at the wound site was observed at day 28, with the SF nanomatrix-treated group exhibiting a relatively denser and more continuous collagen array compared to the control group. It was therefore concluded that the SF nanomatrix amplified burn wound restoration, thus demonstrating its potential use for burn wound treatment [81].

Peifen et al. conducted a 3D scaffold design using sulphated silk fibroin (SSF), chitosan (CS), and hydroxyapatite (HA) materials, highlighting the significance of blood coagulation. The design’s focus on SSFs anti-conjugation behavior relative to SF necessitated the evaluation of co-culture, cytotoxicity, and animal tests. Upon comparing the results, it was found that the SSF/CS/HA and SF/CS/HA groups displayed favorable cytotoxicity conditions, whereas the SSF/CS/HA scaffold group showed a higher statistical outcome on the 7th day of animal experimental application. Consequently, it was concluded that the designed scaffold is biocompatible and biodegradable; it promotes cell viability and proliferation, suggesting its potential for use in wound healing [82].

In addition to previously introduced research, Yin et al. investigated the mechanical enhancement of SF, a well-established material. The hydroxypropyl methylcellulose (HPMC) type of cellulose, which comes in varying configurations, was preferred in this study. The SF/HPMC solution’s viscosity and electrical conductivity were evaluated, and the influence of the SF/HPMC weight ratio was analyzed. To better understand the spinning behavior, SF/HPMC compositions with varying ratios were produced at voltages ranging from 55 to 61 kV. The resulting nanofiber scaffolds were analyzed, and it was found that the SF/HPMC exhibited favorable spinnability [83].

Silk is emerging as a valuable asset in the field of regenerative medicine, yielding promising results through extensive research. For example, Gupta et al. successfully developed a cost-effective and rapid silk solution hydrodynamic concentration method by simulating the internal bending of silk, as observed in a silkworm. To this end, they designed a 3D multilayer microfluidic device with one outlet and three inlets [84].

### 2.3. Silk Fibroin in Bone Tissue Engineering

Bone plays a pivotal role in the human body, with critical tasks ranging from safeguarding internal organs to regulating the body’s pH levels and maintaining homeostasis. Bone tissue is a hard tissue consisting of 35% organic components, including collagen, which makes up nearly 95% of the organic ECM, as well as osteocalcin, osteonectin, hyaluronan, and proteoglycans. The inorganic matrix that makes up 65% of bone tissue comprises HA, carbonate, and other inorganic salts [85]. The replacement of such a large and important structure in the event of damage is also extremely important and critical.

Bone tissue engineering is based on replicating the micro- and nanostructure of natural tissue [86] to restore bone tissue defects and promote osteogenesis using biomaterials. To achieve the best possible regeneration akin to the original state, it is imperative to establish the extent of cell damage within the affected zone, comprehensively assess the damage severity, and review and appraise treatment approaches according to unique circumstances. The primary concern with these approaches lies in selecting biomaterials with optimal biocompatibility and minimal toxicity for bone applications [85]. To mitigate such risks, various techniques and materials have been employed for the regeneration and repair of bone tissue. HA, SF, and gelatine are among the several materials utilized.

Recently, there has been a rapid increase in the use of silk fiber in bone tissue engineering. Its strong mechanical properties, including strength of 0.6 GPa, extensibility of 0.8, and toughness of 70 mJm^−3^ [86], which is a crucial feature for bone [87], make it an appealing material for bone tissue engineering [2]. Fixation devices, including SF-based screws, plates, and K-wires, have the advantage of being resorbable and biocompatible. They also offer significant therapeutic benefits, including the ability to promote a minimal inflammatory response and even stimulate bone repair [88].

In addition to mechanical strength and biocompatibility, the antimicrobial effect plays a crucial role in bone repair studies. It is undeniable that bacterial resistance poses a significant obstacle to complete bone repair. Ribeiro et al. incorporated silver nanoparticles (AgNPs) to improve the antimicrobial effect of the fabricated SF/nHA hydrogel. The study revealed that SF/nHA hydrogel containing substantial amounts of AgNPs had the ability to decrease the number of strains of both gram-positive (*S. aureus*) and gram-negative (*E. coli*) bacteria [89].

Jo et al. utilized SF alongside alginate and HA for bone replacement and examined their behavior in vivo. Notably, it was observed that the alginate/HA/SF scaffold demonstrated significantly greater bone formation at the affected site approximately one month after implantation compared to the control group (*p* = 0.044) and the alginate scaffold (*p* = 0.035). Similarly, the study’s outcomes also highlighted the scaffold’s high biocompatibility and reduced immunogenic response in the defective area. Thus, the scaffold was presented as a promising, biodegradable bone structure for rats [90].

Sheikh et al. created and characterized a hybrid scaffold that consisted of PLGA (75:25 lactide:glycolite), silk, and HA NPs. The fabricated scaffolds were tested in vivo, and the obtained results indicate that the control sample exhibited no observable signs of bone formation. In contrast, the pristine silk and PLGA scaffolds demonstrated the least and moderate levels of bone formation, respectively. The PLGA-silk scaffold exhibited higher bone formation compared to either of the individual components. Notably, the PLGA-silk-HA scaffolds displayed the highest amount of bone formation. These findings aligned consistently with the results obtained from both MTT assays and histological examination, advancing the PLGA-silk-HA scaffolds as excellent candidates for tissue engineering applications [91].

Du et al. combined bioactive glass (BG), a noteworthy material in hard tissue engineering, with a functional natural polymer (i.e., SF). A mesoporous bioactive glass (MBG)/SF composite scaffold was 3D-printed for use in bone tissue engineering applications. According to the study, the optimal mass proportion of MBG and SF solution was determined to be 80%:20%. Thus, the hybrid scaffold exhibited superior compressive strength, remarkable biocompatibility, and bone-forming ability in comparison to the control groups. A novel method was therefore employed to develop structures with remarkable osteogenic capabilities and mechanical properties, making them well-suited for bone applications [92].

Gambari et al. introduced a novel technique to enhance bone tissue proliferation in vitro. Specifically, they loaded GYY4137, a hydrogen sulfide (H_2_S) donor known for its positive effect on bone tissue, onto SF scaffolds. The GYY was prepared using two concentrations, 1.25 and 5%, and released slowly over time. Based on these findings, it has been determined that the combination of SF and H_2_S activates genes and proteins that contribute to both bone mineral metabolism and angiogenesis, resulting in accelerated and increased mineralization. It was therefore concluded that the increase in bone cell growth was achieved by loading SF scaffolds with the H_2_S donor GYY4137 [93].

Zheng et al. utilized a composite of SF, calcium silicate (CS), and sodium alginate (SA) to regenerate bone tissue. Structural, chemical, and thermal characterization assessments of the SF/CS/SA scaffold, along with in vitro investigations, were conducted. Osteogenic differentiation tests indicated a significant difference (*p* < 0.05) between the pristine culture solution (control) and the fabricated scaffold. Thus, the alkaline phosphatase activity values inferred for the bone marrow mesenchymal stromal cells were superior in the case of the SF/CS/SA scaffold (~73) as compared to the control (~58). The same significant difference between the two analysis groups was also indicated in the case of cell viability tests. The results of this study should therefore advance the SF/CS/SA composite scaffold as a biocompatible solution for the repair of bone tissue defects and injuries [94].

Mobika et al. manufactured HA/SF nanocomposite structures. A 6% SF aqueous solution was prepared using formic acid and crosslinked with glutaraldehyde. It was observed that as the proportion of SF increased, the crystallinity in the nanocomposite structure decreased. This result led to an increase in cell proliferation and integration. To form the composite structure with HA, the co-precipitation method was used. Through this method, SF provided biodegradability and biomineralization properties to the scaffolds while maintaining their secondary structure. The study successfully mimicked the ECM of bones, resulting in the production of a cytocompatible scaffold [95].

The research conducted by Wei et al. focused on the development of a composite bio-ink composed of SF, gelatine, hyaluronic acid, and tricalcium phosphate. Hybrid scaffolds using SF were manufactured using extrusion-based 3D bioprinting processes. The 3D bio-printed scaffolds underwent double crosslinking and were later exposed to human platelet-rich plasma (PRP) to form PRP-coated scaffolds. Live/dead and MTT analyses demonstrated that PRP treatment significantly promoted cell growth and proliferation of human adipose-derived mesenchymal stem cells. The study revealed that 3D printing of 6.8% SF-based hybrid scaffolds, combined with post-treatment PRP, may represent a more effective approach to support osteogenic differentiation of adult stem cells and, thus, hold significant potential for bone tissue engineering applications [96].

### 2.4. Silk Fibroin in Cartilage Tissue Engineering

Cartilage tissue engineering (CTE) is a technique deployed to treat defects resulting from age-related problems, such as osteoarthritis or trauma [97]. Like others, CTE is a complex procedure that necessitates the fabrication of 3D scaffolds to be effective. To treat and address CTE injuries, the goal is to induce chondrogenic differentiation using appropriate biochemical agents [98]. The 3D scaffold should be porous to provide a favorable environment for chondrogenic cells and to facilitate the formation of cartilage-specific ECM. The selection of the polymer to be used for this purpose is therefore crucial. SF represents one potential option that is often employed [99,100]. Zhou et al. produced photo-cross-linkable Sil-MA by functionalizing silica. Next, a micro/nano hydrogel based on chitosan and Sil-MA at various concentrations was developed. The compressive modulus for the hydrogel with 0.1% Sil-MA was determined to be 0.32 ± 0.07 MPa, which was consistent with previous literature data. Additionally, it was demonstrated through in vitro cytotoxicity and in vivo (mouse) experiments that these hydrogels are strong contenders that could be utilized for cartilage repair [101].

### 2.5. Silk Fibroin in Drug Delivery Systems

SF has a crucial role in drug delivery as a carrier for biomacromolecules, owing to its impressive biocompatibility, biodegradability, and low immunogenicity. SF NPs are customizable with high binding capacity, controlled drug release properties, and gentle preparation conditions for a wide range of medical applications. By adjusting the particle size, chemical structure, and properties, SF-based NPs have the potential to deliver a range of drugs, including small-molecule drugs (such as those used in cancer treatment), protein and growth factor drugs, and gene drugs, among others [102,103].

NPs possess the ability to overcome physiological barriers and pass through small capillaries, enabling their incorporation into cells. Thus, silk-based nanospheres are currently under investigation for their potential in drug delivery, particularly for treating various ailments, including cancer. Preliminary clinical findings demonstrated that nanoparticle therapeutics offer greater effectiveness in comparison to free chemotherapeutics and have reduced side effects due to their properties, including tumor-specific localization and active cellular uptake [86].

## 3. Advantages of Silk-Based Hydrogels in Tissue Engineering

Silk-based hydrogels have emerged as a viable biomaterial for various biomedical applications due to their unique combination of biocompatibility, mechanical strength, and tunable characteristics. SF, a natural protein isolated from silkworm silk, is the primary source of silk-based hydrogels [104]. These hydrogels have several distinct characteristics that make them an appealing alternative for biomedical research and applications. Silk-based hydrogels are biocompatible because SF is non-immunogenic and does not elicit negative immune responses [105]. This biocompatibility is crucial for tissue engineering applications, where scaffold materials need to support cell adhesion, proliferation, and tissue regeneration. Secondly, silk-based hydrogels have demonstrated superior mechanical characteristics. They can be designed to match the mechanical properties of specific tissues, offering support while preserving structural integrity [106]. This mechanical adaptability is useful in tissue engineering and can assist in the creation of biomimetic scaffolds for a variety of tissues, such as bone, cartilage, and vascular tissue. Silk-based hydrogels also have regulated drug delivery capabilities. Their porous structure allows for the therapeutic chemicals to be incorporated and released over time, making them ideal for localized drug delivery applications. This property has great promise in cancer therapy, wound healing, and regenerative medicine [107]. Their inherent biocompatibility and controlled drug delivery properties contribute to enhanced wound healing results. Moreover, silk-based hydrogels can be changed and functionalized with imaging agents, allowing them to be used in bioimaging applications. Due to their versatility, they represent important tools for non-invasive imaging, aiding diagnoses, and disease progression monitoring [108].

## 4. Limitations of Silk-Based Hydrogels in Tissue Engineering

Although silk-based hydrogels have received great attention for their potential properties in biomedical applications, it is critical to acknowledge their inherent drawbacks to make informed decisions about their suitability for specific situations. One notable disadvantage is that they degrade at a slower rate than some other biodegradable polymers [109]. This situation may inhibit the natural turn-over of tissue scaffolds, possibly complicating applications where the hydrogel is intended to be resorbed as new tissue grows. Another drawback of silk-based hydrogels is that they can be immunogenic if not sufficiently purified. Residual sericin, a protein contained in raw silk, can cause immunological reactions in some people [110]. Due to its immunogenic potential, stringent purification techniques are required, which can add complexity and cost to the hydrogel manufacturing process. Furthermore, silk-based hydrogels may not represent the best solution for some drug delivery applications that require rapid and accurate release kinetics. While the sustained release profile of silk-based hydrogels may be beneficial in some situations [111], it may be less desirable when immediate and regulated drug release is required. To summarize, while silk-based hydrogels have various advantages, they also have significant limitations, such as slow degradation rates, possible immunogenicity, and restricted appropriateness for specific drug delivery applications. When considering the suitability of silk-based hydrogels for biomedical applications, these constraints must be carefully considered, as they may not be the best option in all circumstances.

Last but not least, considering its high crystallinity and hydrophobicity due to the large number of hydrogen bonds, it is important to mention that SF is insoluble in most organic solvents and water [112].

## 5. Future Aspects and Challenges

The conformation, morphology, and Young’s modulus of SF-based hydrogels have positioned them over the years as valuable candidates for various tissue engineering applications. Nevertheless, the numerous applications of SF-based hydrogels across different fields have brought to light some inadequacies. Among these, one could mention the varying silk protein manufacturing techniques and conflicting degumming processes [88]. As such, there is a constant need to optimize this complexity. The use of HA in composite hydrogels with SF for bone tissue engineering is a widely adopted practice. Nonetheless, further in-depth research, experimentation, and testing are still necessary for a comprehensive understanding of the HA structure at the micro-nano-scale. Numerous studies have also highlighted the advantages of employing nHA-SF scaffolds for bone tissue engineering, both in vitro and in vivo. Nevertheless, it should be emphasized that a comprehensive investigation regarding their potential negative impact is currently missing. Despite the beneficial characteristics of SF/HA in this area, it is crucial to design efficacious constructs that can be utilized clinically. It should be noted that numerous studies have verified the osteogenic potential of SF scaffolds in vivo; however, their applicability to human use may not be fully extrapolated from animal models [90]. Overall, from the reviewed literature, silk products emerged as promising candidates for a wide range of bone tissue engineering applications.

Furthermore, there is a restricted use of Sil-MA as a novel commodity in the literature, which consequently leads to an incomplete understanding of its characteristics. But with regards to silk-based hydrogels, Sil-MA has the potential to be a revolutionary advancement.

## 6. Conclusions

This review examines some applications of silk fibroin (SF) and its derivatives in sub-branches of tissue engineering. Thus, the results reported and briefly analyzed in this review indicate that SF is successfully utilized as a carrier in bone regeneration, treatment of cartilage defects, wound healing, and drug delivery systems. However, one should stress that the rapidly increasing number of various research studies on SF is a testimony to the continuous efforts to use this spectacular biomaterial in application areas other than tissue engineering. Overall, the selected literature suggests that silk-based hydrogels are a promising avenue for further in-depth studies, with extensive in vitro and animal tests still necessary.

## Data Availability

Not applicable.
